# The *incC* Sequence Is Required for R27 Plasmid Stability

**DOI:** 10.3389/fmicb.2016.00629

**Published:** 2016-05-03

**Authors:** Eleonora Tassinari, Sonia Aznar, Imanol Urcola, Alejandro Prieto, Mário Hüttener, Antonio Juárez

**Affiliations:** ^1^Institut de Bioenginyeria de CatalunyaBarcelona, Spain; ^2^Departament de Microbiologia, Facultat de Biologia, Universitat de BarcelonaBarcelona, Spain

**Keywords:** IncHI1 plasmids, plasmid R27, plasmid stability, antimicrobial resistance, *incC*, E protein

## Abstract

IncHI plasmids account for multiple antimicrobial resistance in *Salmonella* and other enterobacterial genera. These plasmids are generally very stable in their bacterial hosts. R27 is the archetype of IncHI1 plasmids. A high percentage of the R27-encoded open reading frames (ORFs) (66.7%) do not show similarity to any known ORFs. We performed a deletion analysis of all non-essential R27 DNA sequences to search for hitherto non-identified plasmid functions that might be required for plasmid stability. We report the identification of a short DNA sequence (*incC*) that is essential for R27 stability. That region contains several repeats (*incC* repeats), belongs to one of the three-plasmid replicons (R27 FIA-like) and is targeted by the R27 E protein. Deletion of the *incC* sequence drastically reduces R27 stability both in *Escherichia coli* and in *Salmonella*, the effect being more pronounced in this latter species. Interfering with *incC*–E protein interaction must lead to a reduced IncHI1 plasmid stability, and may represent a new approach to combat antimicrobial resistance.

## Introduction

Resistance to antimicrobial compounds (AMR) is nowadays considered as a major global threat to public health (WHO, 2014). Plasmids play a key role in the dissemination of AMR, since they facilitate the spread of the resistance determinants, can be positively selected by antimicrobial compounds, and are difficult to cure or to counteract by the available strategies (Carattoli, 2013). Novel Eco–Evo strategies to combat AMR take into account that AMR is widespread in the nature and are focused to prevent the evolution and emergence of resistant bacteria in the environment (Baquero et al., 2011). The goal of these new approaches is not to kill multiresistant microorganisms, but to prevent their emergence or evolution, or to reestablish the antibiotic susceptible populations. In this context, controlling persistence and dissemination of resistance plasmids (R plasmids) is considered as a key strategy to fight AMR (Baquero et al., 2011). In the absence of selection, persistence relies on the ability of the plasmids to be stably maintained in their hosts, and in the ability of these genetic elements to be transmitted by horizontal gene transfer mechanisms. Acquisition of a plasmid may result initially in a fitness cost to the host (Dahlberg and Chao, 2003). The reasons behind plasmid biological cost remain poorly investigated. It is known that such a cost frequently peaks after the acquisition of a novel plasmid for the host organism, and the cost is progressively reduced during coexistence time, in a kind of “plasmid domestication” (Baquero et al., 2011). Domestication probably results from a complex rewiring of the cell metabolism involving the presence of the plasmid.

Plasmid cost frequently increases upon expression of the resistance gene(s) (for instance, after transcriptional induction), and this cost is variable for different genes (Nogueira et al., 2009). Plasmid biological cost is the reason why large plasmids are always present in low copy numbers in their bacterial hosts. Otherwise, the metabolic burden of maintaining and duplicating their genomes would be excessive. Therefore, mechanisms that ensure faithfully transmission to daughter cells during cell division are required (Nordström and Austin, 1989). Mechanisms underlying plasmid stability include partitioning, multimer resolution and post-segregational killing (Sengupta and Austin, 2011). In spite of the presence of these mechanisms, some plasmids can be lost at detectable frequencies in the absence of selective pressure. On the other hand, several plasmids encoding AMR are extremely stable in their bacterial hosts. Hence, it is relevant to investigate novel mechanisms influencing plasmid stability as a means to design new strategies to combat antimicrobial resistance.

Plasmids of the incompatibility group IncHI are widespread in the *Enterobacteriaceae*. Within the genus *Salmonella*, they account for a significant proportion of antibiotic resistance in the most common invasive *Salmonella* serovars: *S*. *enterica* serovar Typhi and *S*. Paratyphi A (Holt et al., 2011). With respect to *S*. Typhi, antimicrobial resistant typhoid has been observed for half a century and is now common in many areas. IncHI plasmids are prevalent in *S.* Typhi: more than 40% of all isolates harbor an IncHI plasmid (Holt et al., 2011). In the last years, IncHI plasmids encoding extended-spectrum beta-lactamases (EMBLs that hydrolyze cephalosporins) and carbapenemases (the antibiotic choice to combat strains expressing EMBLs) have also been isolated in other enterobacteria such as *Citrobacter* or *Klebsiella* (Villa et al., 2012; Dolejska et al., 2013).

Regulation of conjugative transfer of IncHI plasmids shows a distinctive feature: transfer is repressed at temperatures within the host (37°C) and induced at temperatures outside the host (22–30°C; Maher et al., 1993). Plasmid R27 is the prototype of IncHI plasmids. It encodes resistance to tetracycline (Tc) only and has been intensively studied for over 20 years. R27 replication and conjugation determinants are well characterized (Lawley et al., 2002; Alonso et al., 2005) and its complete nucleotide sequence is available (Sherburne et al., 2000). IncHI plasmids share a common core of about 160 kbp. The differences in size are due to the distinct presence of insertion elements (Gilmour et al., 2004). It displays two features that are shared by many R plasmids: (i) When present in its host (*Salmonella*), R27 is fairly stable, and (ii) a very significant percentage of the R27-encoded open reading frames (ORFs) (66.7%) do not show similarity to any known ORFs. As a means to elaborate a strategy to reduce R27 stability in *Salmonella*, we decided to investigate if any of the ORFs of unknown function plays a role enhancing plasmid stability.

## Materials and Methods

### Bacterial Strains and Plasmids

All bacterial strains and plasmids used in this work are listed in **Table 1**. Oligonucleotides for PCR amplification are listed in **Supplementary Table S1**. Except for conjugation experiments, cultures were grown in Luria Bertani (LB) broth (10 g l^-1^ NaCl, 10 g l^-1^ tryptone, and 5 g l^-1^ yeast extract) with vigorous shaking at 200 rpm (Innova 3100, New Brunswick Scientific). SOB medium (20 g l^-1^ Peptone, 5 g l^-1^ yeast extract, 0.58 g l^-1^ NaCl, 0.19 g l^-1^ KCl, and 20 mM MgCl_2_ + MgSO_4_ per liter; Hanahan et al., 1991) was used to grow bacteria for the inactivation gene experiments. SOC medium (SOB medium with 20 mM glucose) was used to recover cells after electroporation during gene inactivation experiments. Penassay broth (PB; 1.5 g l^-1^ beef extract, 1.2 g l^-1^ yeast extract, 5 g l^-1^ peptone, 1 g l^-1^ glucose, 3.5 g l^-1^ NaCl, 1.32 g l^-1^ KH_2_PO_4_, and 4.82 g l^-1^ KHPO_4_⋅3H_2_O) was used to perform conjugal transfer. MacConkey agar was used to distinguish *S. enterica* serovar Typhimurium from *Escherichia coli* cells upon conjugation.

**Table 1:** Bacterial strains and plasmids used in this work.

Strain	Relevant characteristics	Reference or source
*Escherichia coli* MG1655	F-, ilvG, rph1	Guyer et al., 1981
BL21DE3	T7 polymerase upon IPTG induction	Studier and Moffatt, 1986
SL1344	*Salmonella enterica* serovar Typhimurium SL1344 *his*^+^	Gulig and Curtiss, 1987

**Plasmid**	**Description**	**Reference and provenience**

pKD3	oriRγ, Cm^r^, and Ap^r^	Datsenko and Wanner, 2000
pKD46	oriR101, repA101 (ts), AraBp-gam-bet-exo (Red helper plasmid, Ts; Apr)	Datsenko and Wanner, 2000
R27	IncHI1, Tc^r^	Grindley et al., 1972
pLATE31	Bacterial expression of proteins with C-terminal 6x His-tag	Thermo Scientific

When necessary, the media were supplemented with 10 mM L-arabinose or with the following antibiotics: ampicillin (100 μg ml^-1^), chloramphenicol (25 μg ml^-1^), or Tc (15 μg ml^-1^).

### Genetic Manipulations

All enzymes used to perform standard molecular and genetic procedures were used according to the manufacturer’s recommendations. To introduce plasmids in *E. coli*, bacterial cells were grown until a OD_600__nm_ 0.6–0.8. Then, cells were washed several times with 10% glycerol, and the respective plasmids and PCR-amplified DNA fragments were introduced by electroporation (1,250 V) using an *Eppendorf* gene pulser (Electroporator 2510).

R27 mutant derivatives lacking different DNA fragments in strain *E. coli* MG1655 were obtained by the λ Red recombinase method as described (Datsenko and Wanner, 2000). Briefly, the antibiotic-resistance cassette of chloramphenicol of plasmid pKD3 was amplified by PCR using pairs of about 56–70 nt-long primers, of which 36–50 nt are homologous to the up and downstream regions of the R27 sequences to be disrupted, while the remaining sequences pair up with the P1 or P2 sites on pKD3 plasmid. DNA templates were treated with *Dpn*I (Thermo Scientific) following manufacturer recommendations, and then, purified and electroporated to the competent *E. coli* cells harboring plasmids pKD46 and R27. Mutants were selected on LB plates containing the appropriate selection marker (chloramphenicol in this case) and the successful deletion of the desired R27 fragment was confirmed by PCR.

### Plasmid Stability Assay

Plasmid stability has been determined upon seven successive subcultures at a ratio of 1:10.000 of the plasmid-carrier strain on LB medium (unless otherwise indicated, at 37°C) without antibiotic. After the first and the last overnight cultures, aliquots were diluted and spread on LB agar plates. The isolated colonies were picked in duplicate to LB agar plates with and without antibiotic. Tc-sensitive clones were confirmed for plasmid loss and not for Tn*10* loss by PCR. Oligonucleotides Del2_up and Del2_Inner (see **Supplementary Table S1**) amplify a 524 bp region of R27. These oligonucleotides do not amplify chromosomal DNA. Plasmid stability is expressed as the percentage of colonies, which maintain the plasmid, and it is determined as the ratio between the number of antibiotic-resistant isolates and the total number of colonies replicated on LB medium lacking the antibiotic.

### Bioinformatic Analysis

To search for similarities of the 322 bp sequence included in deletion DelNCD, the blastN algorithm was used (Altschul et al., 1990). The results were filtered and only hits with an identity of higher than 95% and coverage of higher than 90% were considered as positive.

### E Protein Purification

The recombinant plasmid for C-terminal His-tagged R27-encoded E protein overexpression was obtained following the instructions of ALICator Ligation Independent Cloning and Expression System (Thermo Scientific) using primers RepECtermFor and RepECtermRev (**Supplementary Table S1**). Recombinant clones were sequenced before overexpression.

Transformed BL21(DE3) cells were grown at 37°C in LB until the cultures reached an OD_600nm_ of 0.5–0.6. Isopropyl-β-D-1-thiogalactopyranoside (IPTG) was subsequently added to a final concentration of 1 mM. Upon 3 h incubation at 37°C, cells were centrifuged and pellets were frozen, resuspended in lysis buffer (50 mM NaH_2_PO_4_ pH 8, 1 M KCl, 10 mM imidazole suplemented with 1 mg ml^-1^) of lysozyme and a protease inhibitor cocktail (cOmplete ULTRA Tablets Mini, EDTA-free, EASYpack Roche) and disrupted by sonication on ice. The lysate was centrifuged at 20,000 × *g* for 30 min at 4°C, and the supernatant was then treated with Ni-NTA (Ni^2+^-nitrilotriacetate)–agarose (Qiagen). The resin was washed extensively with the same buffer, and the protein was eluted using 200 mM imidazole as described previously (Nieto et al., 2000).

### Band Shift Assays

Band shift assay were performed in the presence of radiolabeled *incC* DNA together with increasing concentrations of E protein-his. A 235 nt fragment corresponding to the R27 *incC* sequence (nucleotide positions 156,644–156,878 – NCBI accession number NC_002305.1) was generated by PCR using primers incCFor/incCRev (**Supplementary Table S1**) and gel-purified. Ten picomoles of the PCR product was radiolabeled with [γ-32P] ATP (PerkinElmer) using T4 Polynucleotide Kinase (ThermoScientific) according to the manufacturer’s instructions. For each reaction, 20 fmol DNA template was mixed with 200 ng of poly(dI–dC) and increasing concentrations of E protein-his (0.24–3.84 μM) in binding buffer (250 mM 4-(2-hydroxyethyl)-1-piperazineethanesulfonic acid (HEPES), pH 7.4, 350 mM KCl, 5 mM EDTA, 5 mM dithiothreitol (DTT), 500 μg BSA ml ^-1^, and 25%, v/v, glycerol) and incubated at room temperature for 30 min. The samples (20 μl) were separated on a 5% polyacrylamide/0.5× Tris-Borate-EDTA (TBE) gel. The bands were visualized using Quantity One software (Bio-Rad).

## Results

### Construction of R27 Plasmid Deletions

**FIGURE 1:**
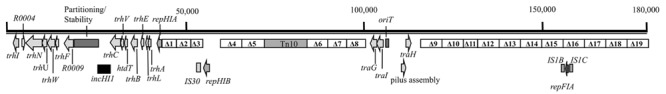
**R27 physical map.** The 19 deletions attempted are shown (Δ1–Δ19).

Deletions in plasmid R27 were performed in the host strain *E. coli* MG1655. Deletion length was about 5–7 kbp. Deletions covered the complete sequence of plasmid R27, leaving out the two main replicons, the regions involved in plasmid stability/partitioning and in plasmid conjugation and the Tc resistance determinant (Tn*10*). The overall number of deletions attempted is 19. Every deletion includes several ORFs whose function in many instances is unknown, and only their similarity percentage with proteins of known function has been annotated. Plasmid location, precise length, and the genes included in each deletion are listed in **Supplementary Table S2**. **Figure 1** summarizes the deletions performed in the R27 sequence.

Upon screening, several positive colonies were obtained for each deletion except for deletion 18 which, was unsuccessfully attempted several times. A single colony containing each of the different R27 derivatives (deletions 1–17 and 19), was purified, checked by PCR, and used to study plasmid stability. Only R27 derivative harboring deletion 16 showed a very significant decrease of stability (85%). We therefore decided to identify the DNA sequences included in the DNA fragment deleted in plasmid R27Δ16 that play a role in R27 stability in strain MG1655.

### *incC* Repeats Are Required for R27 Plasmid Stability in *E. coli* Strain MG1655

**FIGURE 2:**
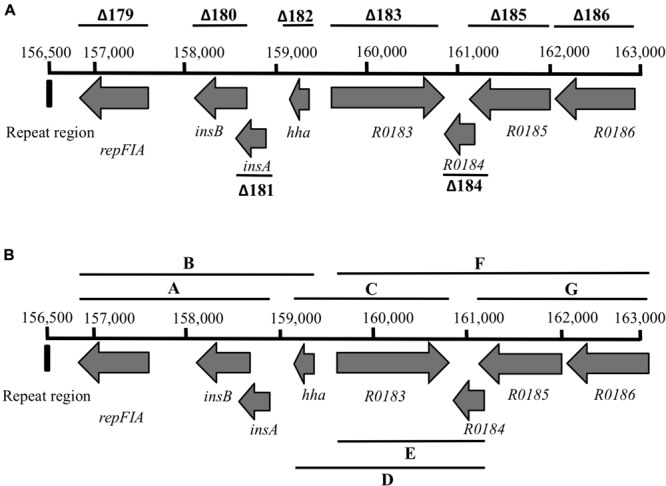
**Physical map of the 6.5-kb DNA fragment replaced in deletion 16. (A)** Deletions performed affecting each of the ORFs included in the fragment. **(B)** Combined deletions in the 6.5-kb DNA fragment.

To identify the DNA sequences whose deletion reduces R27 plasmid stability, a fine mapping analysis was performed. The 6.5-kb DNA fragment replaced in deletion 16 (**Figure 2**) includes eight ORFs, some of which share an extended homology with known proteins, as in the case of ORFs R0179, R0180, R0181, and R0182. The rest do not show significant similarity to other identified ORFs (see **Supplementary Table S2**). We initially hypothesized that any of the single ORFs encoded in the deleted DNA fragment should account for plasmid loss. To confirm this, single ORF deletion mutant derivatives of plasmid R27 were constructed in strain MG1655 using the same mutagenesis protocol previously described. The single deletions were designed at the exact ends of the corresponding ORFs (**Supplementary Table S2**; **Figure 2A**). Plasmid stability assays were performed with the eight R27 derivatives lacking each of the eight ORFs included in the DNA fragment. No single deletion accounted for the previously observed reduced R27 stability (data not shown). We hypothesized then that plasmid instability of R27Δ16 variant being caused by a combined effect of more than one of the corresponding ORFs. To confirm this, we performed deletions combining different ORFs (see **Supplementary Table S2**; **Figure 2B**). The resulting R27 derivatives were tested for plasmid stability. Again, no effect was observed (data not shown).

The unique DNA fragment not included in the combined deletions of fragment R27-16 is a 348-bp sequence located in its 5′ end (**Figure 2**). This non-coding DNA region includes a 25-nt-long fragment of IS1b 3′-end (an insertion sequence) while the remainder of the sequence is not annotated on the previously published R27 map (Sherburne et al., 2000). As this sequence had hitherto not been included in the partial deletions constructed within the DNA fragment corresponding to deletion 16, we performed a new deletion in plasmid R27, spanning from the exact beginning of the DNA sequence corresponding to deletion 16 end, to ORF R0182 (*hha*) included (**Figure 2**). The new plasmid derivative, R27 Δ16P1-182, exhibited high plasmid instability (23% plasmid loss). As this latter large deletion included the 348 nucleotides upstream ORF R0179 and confers plasmid instability, we hypothesized that deletion of the 348-bp DNA fragment, either alone or in combination with any ORF included in deletion 16P1-182 accounts for the observed plasmid instability. To clarify this, a new deletion in R27 was obtained spanning from nucleotides 156,500–156,890 (**Figure 2**). The mutant plasmid obtained (R27ΔNCD) was then analyzed for plasmid stability. Plasmid loss was of 38%.

**FIGURE 3:**
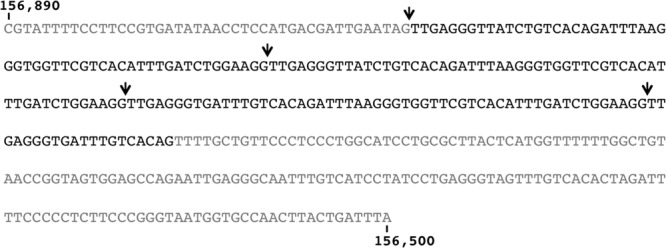
***incC* region.** Arrows point to the start and end of the different *incC* repeats. Note that the last *incC* repeat is truncated.

Upon having confirmed that deletion of a short non-coding region comprised between R27 nucleotides 156,500 and 156,890 significantly reduces R27 stability, the sequence was blasted to search for similarities with already characterized DNA sequences. We used for the analysis the 322-bp sequence within deletion DelNCD which is not annotated on R27 map (Sherburne et al., 2000). The results obtained (**Supplementary Table S3**) show that the sequence aligns completely and for its whole length with sequences present in several other plasmids, mainly isolated from the *Enterobacteriaceae*. Remarkably, that sequence is present in IncHI as well as in IncF plasmids. It belongs to the IncFIA-like replicon of R27 (Saul et al., 1988), and corresponds to *incC* (**Figure 3**). It has been shown that that this sequence includes directed repeats that are targeted by the E protein (Saul et al., 1988).

## The *incC* Sequence Is Required for High Stability of R27 Plasmid in *E. coli* and *Salmonella*

**FIGURE 4:**
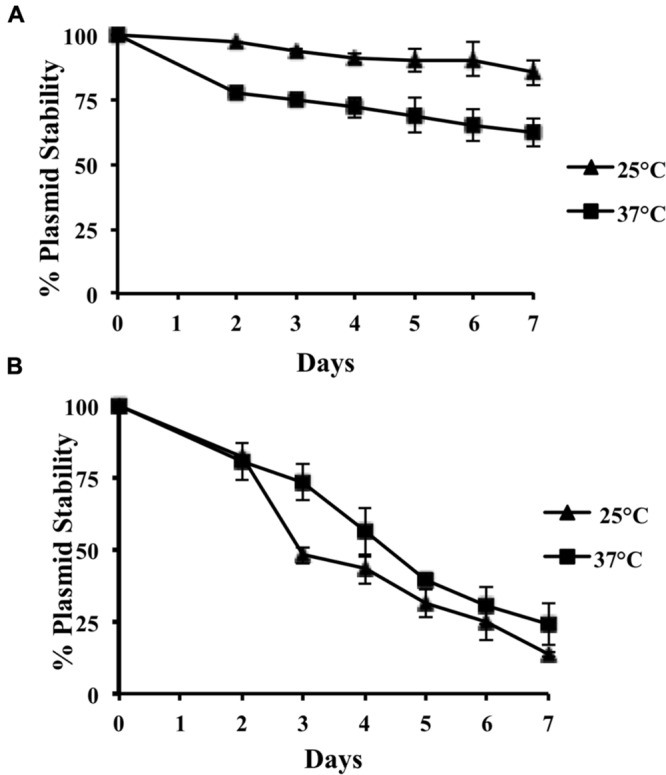
**Stability of R27ΔNCD in *E. coli* MG1655 **(A)** and *S.* Typhimurium SL1344 **(B)** growing at 25 and 37°C, respectively.** The stability of wt R27 in these strains at both temperatures is of 100% upon seven subcultures.

We decided next to more precisely determine the role of the *incC* sequence in plasmid stability both in *E. coli* and *Salmonella.* For plasmid stability studies in *E. coli*, strain MG1655 was used. We compared plasmid stability at 25 and 37°C along 100 generations. MG1655 cells harboring wt R27 do not lose the plasmid at a detectable frequency. When hosted in strain MG1655, R27 *incC* sequence plays a relevant role in plasmid stability when cells grow at 37°C, but not when cells grow at 25°C (**Figure 4A**). Taking into account that IncHI plasmids are predominantly isolated from *Salmonella* strains, we decided to test the role of *incC* in this microorganism. Plasmid R27ΔNCD was conjugated to *S.* Typhimurium strain SL1344 and transconjugants were then used to analyze plasmid stability both at 37 and 25°C (**Figure 4B**). Remarkably, both at 37 and 25°C, the role of *incC* on plasmid stability is even more relevant in strain SL1344 than in strain MG1655. In strain SL1344, R27ΔNCD plasmid loss occurs at higher frequencies; being this case, the effect of temperature is negligible.

### R27-Encoded E protein Binds the *incC* Sequence

**FIGURE 5:**
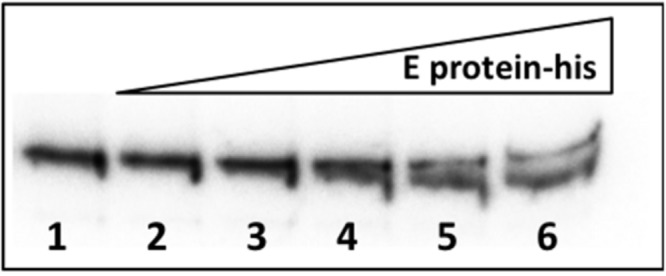
**Band shift assay of the R27 E protein with a DNA fragment including the *incC* sequences.** Reactions were performed in presence of *incC* DNA, 20 fmol, together with 200 ng of Poly(dI–dC) for each reaction, and increasing concentration of E protein-his. Lane 1, No protein; lane 2, 0.24 μM; lane 3, 0.48 μM; lane 4, 0.96 μM; lane 5, 1.92 μM; and lane 6, 3.84 μM.

It was indirectly shown that R27 *incC* sequences were able to bind F plasmid-encoded E protein (Saul et al., 1988), but no direct evidence of R27 E protein binding *incC* sequences has hitherto been provided. To show this, we purified his-tagged R27 E protein and performed band shift assays with a 235-bp DNA fragment including the *incC* sequences. As predicted, low mobility protein–DNA complexes are obtained upon incubating E protein with the DNA fragment (**Figure 5**).

## Discussion

IncHI1 plasmids such as R27 contain three replicons (RepHI1A, RepHI1B, and RepFIA-like). Previous studies showed that deletion of the RepFIA sequences from R27 leaves a plasmid, which is both compatible with F and replication-proficient. Each of both RepHI1 replicons appears to be capable of taking over the replication of the plasmid (Gabant et al., 1993). The deletion analysis we performed in plasmid R27 allowed us to identify sequences that map within R27 RepFIA sequences and are required for R27 plasmid stability. Remarkably, the R27 *incC* region whose deletion leads to a marked plasmid instability corresponds to the less conserved region of the RepFIA replicon in R27. When compared to the corresponding region in F plasmid, *incC* repeats are conserved, but not the spacer regions (Saul et al., 1988). R27 RepFIA region was shown to contain a functional E protein, able to repress the E promoter and to initiate replication from F *ori2*. R27 *incB* and *incC* sequences were also shown to bind F plasmid-encoded E protein. RepFIA region of plasmid R27 was considered as responsible for the incompatibility of R27 and F plasmids (Saul et al., 1988). Although all three IncHI1 replicons were identified several years ago (Saul et al., 1988; Gabant et al., 1993, 1994; Newnham and Taylor, 1994) the reasons for the simultaneous presence of all three replicons in this plasmid Inc group remain to be elucidated, as well as their specific biological role. It has been suggested that the carriage of multiple, independent replicons allow plasmids to coexist with other competing plasmids within the same bacterial cell (Gabant et al., 1993).

The results reported here add new information about the biological role of sequences belonging to the RepFIA replicon of R27. Although it has been reported that the either RepHI1A and RepHI1B replicon can overtake R27 replication (Gabant et al., 1993), the results we obtained show that the *incC* region itself plays a critical role in R27 stability, both in *E. coli* and in *Salmonella*. In the absence of *incC*, replication initiation from the two other alternative replicons appears to be less efficient. If one assumes that the observed effect of *incC* sequences on plasmid stability is due to protein E–*incC* interaction, different hypotheses can explain this. In the wt system, protein E–DNA interaction could either titrate the replication initiation protein, hence reducing the replication potential, or promote plasmid replication. Hence, interference with protein E–DNA interaction can either increase plasmid copy number and plasmid stability, or interfere with plasmid replication and reduce plasmid stability. As well, a hypothesis to explain the observed effect is that excess of free-E protein might result in handcuffing interactions between Rep proteins bound at ori sequences.

The effect of R27 *incC* deletion is host-dependent: whereas *incC* deletion has a moderate effect on R27 stability in *E. coli*, deletion of these repeats has a very relevant effect on R27 stability in *Salmonella*. Growth temperature also appears to influence R27 stability in plasmid derivatives lacking *incC* sequences in *E. coli*: reduced plasmid stability can be significantly observed at 37°C, but it is almost neglectable at 25°C. The effect of growth temperature is not significant in *Salmonella.* In this host, stability of R27ΔNCD is much lower than that of the wt plasmid both at 37 and at 25°C. These results suggest that carriage of multiple independent replicons not only may allow plasmids to coexist with other plasmids, but to efficiently replicate in different bacterial hosts and under different environmental conditions.

IncHI1 plasmids can be found both in natural *E. coli* and *Salmonella* isolates. Nevertheless, whereas their presence is scarce in the former (Johnson et al., 2007, 2008), they can be frequently isolated in the latter (Holt et al., 2011). As commented above, they play a relevant role in the AMR phenotype of the *Salmonellae*. IncHI plasmids are conjugatively transferred at temperatures of about 25°C (Maher et al., 1993) and, when *Salmonella* cells grow at these temperatures, a sophisticated crosstalk between these plasmids and the host regulatory networks is established. As a result, IncHI plasmids increase the fitness of *Salmonella* cells (Paytubi et al., 2013). Remarkably, under these conditions, the *incC* region plays a critical role in ensuring IncHI plasmids stability.

Although the basis for the pronounced instability of R27 in the absence of *incC* repeats remains to be elucidated, the fact that these sequences are critical for plasmid maintenance in *Salmonella* raises the possibility of targeting them with the aim to reduce IncHI1 plasmid stability in this microorganism and hence, develop a new approach to decontaminate antibiotic resistance. The fact that at least *in vitro* the R27 E protein is able to bind R27 *incC* sequences suggests that molecules able to interfere with this interaction might be candidates to be tested to reduce IncHI plasmids stability.

## Author Contributions

Conceived and designed the experiments: ET, SA, and AJ. Performed the experiments: ET, IU, and AP. Analyzed the data: ET, SA, MH, and AJ. Wrote the manuscript: ET, SA, MH, and AJ. All authors read and approved the final manuscript.

## Conflict of Interest Statement

The authors declare that the research was conducted in the absence of any commercial or financial relationships that could be construed as a potential conflict of interest.

## Supplementary Material

TABLE S1 **Oligonucleotide sequences used in this work**.

TABLE S2 **R27 sequences included in the different deletions**.

TABLE S3 **Results of the BlastN analysis**.
