# Supplementary material for: The incC Sequence Is Required for R27 Plasmid Stability
Source: Front Microbiol. 2016 May 3;7:629. doi: 10.3389/fmicb.2016.00629 (PMC4853401; doi:10.3389/fmicb.2016.00629)
Supplement: TABLE S1 — Oligonucleotide sequences used in this work. [file Table_1.DOCX]

| Oligonucleotide | Sequence 5’- 3’ |
| --- | --- |
| Del1_P1 | CGTGTTGGTCACATCAAAAGGATGTGTTACCTCATTGTTATCACTCAGT  GTAGGCTGGAGCTGCTTC |
| Del1_P2 | CGGTCAGCAGTAAACTGCTTATCCCATTCTTTCTTCTTTTCGTCAGACCAT  ATGA ATATCCTCCTTAGT |
| cat_rev | CCTACCTGTGACGGAAGATC |
| Del1_UP | GCAACCGAGCCAACACTTAA |
| Del1_DOWN | GTCATTGAGCAACGCGACA |
| Del2_P1 | CAGAATACTGGAAGAGGGTCATGGCCCTGACCGATGTACTGTCTGTGAGTG  TAGGCTGGAGCTGCTTC |
| Del2_P2 | CAGCGCATAAATATGCCGTTATGCTTGGTGACAACGCTAAGATAGCATATG  AATATCCTCCTTAGT |
| Del2_UP | ACTGGTGCTATGTCTGCTGT |
| Del2_DOWN | GACCATTCAGTCGCAGCATT |
| Del3_P1 | TTTATTAAAGCAACAGAGTCTTTTGAGAAGATCTGCGGGAAAAATAAAGG  TGTAGGCTGGAGCTGCTTC |
| Del3_P2 | CATGAAATGCACTAACGTCCATACCTGCTGCACACATACGCGGCA  CATATGAATATCCTCCTTAGT |
| Del3_UP | TTACGCTTTGGACGATTGGC |
| Del3_DOWN | CGCTCGTCTGGGTATGAAAG |
| Del4_P1 | GTGTTTACGGTTTATGTCAACGCGGTATGCGCTGGCCGTTGATCTGTGT GTAGGCTGGAGCTGCTTC |
| Del4_P2 | CAGTTTATTAAAAAAAACTGATAATGCCCCGCCGGAGAATTACGCCACAT  ATGAATATCCTCCTTAGT |
| Del4_UP | CTATGGCAGAACTGGAACGC |
| Del4_DOWN | CGCTGAATTACCGGGCATC |
| Del5_P1 | GCAACCGGGAAAAGTAACTTTTCAGCCAGACCATGCCTTCACAGGAAGTGT  AGGCTGGAGCTGCTTC |
| Del5_P2 | CGGGGCAGAATTGGTAAAGAGAGTCGTGTAAAATATCGAGTTCGCACAC  ATATGAATATCCTCCTTAGT |
| Del5_UP | CATCCGGGAAAGGCGATTTT |
| Del5_DOWN | CTTTGGTTGGCAGGTTACGG |
| Del6_P1 | GCAGAATTGGTAAAGAGAGTCGTGTAAAATATCGAGTTCGCACAGT GTAGGCTGGAGCTGCTTC |
| Del6_P2 | CTTCCCGCGTCGCCAGTCGGGCACTTCCGGAACGGTCGCGGATCAGCCATA  TGAATATCCTCCTTAGT |
| Del6_UP | TAAAGCTAGCATGCCAACGG |
| Del6_DOWN | CGGGCGCTATTTTGTATCCA |
| Del7_P1 | CATTCTGTCGTCATCAAGGAAGTACACGATAATCACGTGATTGCCAGCTGT  GTAGGCTGGAGCTGCTTC |
| Del7_P2 | CTCATAAGAATTTCCTTCATAATCGCCAGGCCGGAGCCTGGCTAGGGGTCA  TATGAATATCCTCCTTAGT |
| Del7_UP | TCACTCTCCCGGACCAAAA |
| Del7_DOWN | GTGTTACCGGTGATTCGCTG |
| Del8_P1 | CAACCAAGACCACTTCGTTAAACTCGCCAAACACCTCGCGGCCTGACGTGT  AGGCTGGAGCTGCTTC |
| Del8_P2 | GAATGTGGGTTTAAGCTATTTAAATAAATATCTAAGGAAATTGCATTGACA  TATGAATATCCTCCTTAGT |
| Del8_UP | ATTTCGGACCTGGCCTATCA |
| Del8_DOWN | ACCCGCCGGAAAAGTAACTT |
| Del9_P1 | GAGAAAAGCGTTCATGGAAAGTCTTCGTGGCACAGGTAACTGAGGTCGT  GTAGGCTGGAGCTGCTTC |
| Del9_P2 | GCCCGGGTGACAATGCTGAGCCAAAGCGACATCTTCTAACCCCCAGC CATA  TGAATATCCTCCTTAGT |
| Del9_UP | TTTCTGCATGGGATGATCGC |
| Del9_DOWN | TTGGTAATAACGGAGGATCACC |
| Del10_P1 | CTTCCAGCCCATTGTTGCGTTCTATGAACGTCTGGCCTGAGTGCGTCGTGTA  GGCTGGAGCTGCTTC |
| Del10_P2 | CATAGACCTAATGAGCGTTTTGTATTATAAATCTCACACAAATCTATCATA T  GAATATCCTCCTTAGT |
| Del10_UP | GCTAAGCCGAGGGATGAGTT |
| Del10_DOWN | TAATTGCCCATTAAGCCACGC |
| Del11_P1 | CAGTGTTTATGAAAGATACTGGGAAATAGTTCAGCCAAATAAATGACGTG  TAGGCTGGAGCTGCTTC |
| Del11_P2 | CAAACTCTTCCCTCTATACATAATTCTGATAAGACAGTGGAGCAACTTCATA  TGAATATCCTCCTTAGT |
| Del11_UP | TTCTTCACAAACAGCCAGGT |
| Del11_DOWN | AGAAGCGACGTGGAAAACAG |
| Del12_P1 | CAGTATTGCGCCGAACGTGCCAGCGCGCGATGGTTGTTGAGTCATTGGTGT  AGGCTGGAGCTGCTTC |
| Del12_P2 | CATTTCCATGTCTGGTGGTGCGATAAGGTTCTGATTTTCAGAACCTTTTCA  TATGAATATCCTCCTTAGT |
| Del12_UP | TGTTCGAGAAAATTACCGCTGG |
| Del12_DOWN | CTCACGGCCAGGGATGTAAA |
| Del13_P1 | GAGTATCTTACGTTTATTCAAAATGAAATCTACTCTAGTAAACTTTAAAGT GTAGGCTGGAGCTGCTTC |
| Del13_P2 | CTAAATCCTCTGGGGCATCAAAACCGCGAAACAAAAAGACGACATAACTC ATATGAATATCCTCCTTAGT |
| Del13_UP | AGGTGCTCAATCAAAATTCCG |
| Del13_DOWN | CGAGGAACAATCAAGCAGCT |
| Del14_P1 | CCTGTGGACGCCAGGAATTTAAGTCTATTGCAAATTCCTGGCGGGAGCGT GTAGGCTGGAGCTGCTTC |
| Del14_P2 | GGCTGGCTGCACGCCACCTTTATATTGAAATCCAACAGAGTTGATACACATA  TGAATATCCTCCTTAGT |
| Del14_UP | AAGGACGTAAACTCCTGCGA |
| Del14_DOWN | ATGGCAGTGTTTTCGGGATG |
| Del15_P1 | AATAACAATGGTCGCTTTTAAATATCCCGAAAGGCAATTTTGCATTTGTGT GTAGGCTGGAGCTGCTTC |
| Del15_P2 | CTAGATTTTCCCCCTCTTCCCGGGTAATGGTGCCAACTTACTGATTTACAT  ATGAATATCCTCCTTAGT |
| Del15_UP | CACTTTCCGCAGTCCCATTT |
| Del15_DOWN | AGTGGAGCCAGAATTGAGGG |
| Del16_P1 | CTGATAGAAACAGAAGCCACTGGAGCACCTCAAAAACACCATCATACACGT  GTAGGCTGGAGCTGCTTC |
| Del16_P2 | ATCGACTCTTCAGAAGTCGTTGAAGACGTAACGGTGTTGCGCTGCTTTCAT  ATGAATATCCTCCTTAGT |
| Del16_UP | GTAGCTGAACAGGAGGGACA |
| Del16_DOWN | GCGGAAGGTGCTGTAAAAGT |
| Del17_P1 | CGGTAAAAGAGTAAATCTTCACTGGCCATTCATCACTCCGGTTTCAAT GT GTAGGCTGGAGCTGCTTC |
| Del17_P2 | GCTACCTATTCTGATCAACTTGCAGGAAAGAATGGCGCAGTAGTACATACA  TATGAATATCCTCCTTAGT |
| Del17_UP | CCGGCTGATGGTCATTCAGA |
| Del17_DOWN | GAGTTGCGTTGGTCCAAGAA |
| Del18_P1 | GAAGTGCCCCACAGGGGAGCGACTTCCCCAGTGGGTGGATTAAAAAGTGT  AGGCTGGAGCTGCTTC |
| Del18_P2 | GGGCAAAACGACGCCCGGTTGGGTCGCTTTGCTCCCTGAAGGATTAAGCAT  ATGAATATCCTCCTTAGT |
| Del18_UP | CCAGTACTGCACGAACGTAG |
| Del18_DOWN | TTGCCGAATGAGGTCAGTAAA |
| Del19_P1 | CTTTTTCATACTCGACACGGTAAGTCTTTATCTGAGTCATATATTGCTCGT GTAGGCTGGAGCTGCTTC |
| Del19_P2 | CTGAAGATGTATATCAGTTATACGTCGTCGCAATGGAAGAGTCGTTGGCAT  ATGAATATCCTCCTTAGT |
| Del19_UP | TTTCACCCATGCGACCAAAA |
| Del2_Inner | GACTGAAAGACGGCATAGAC |
| Del179_P1 | GATCAAATGTGACGAACCACCCTTAAATCTGTGACAGATAACCCTCAA GTG  TAGGCTGGAGCTGCTTC |
| Del179_P2 | ATGAAGTGTGACAATCTTAAAGTCTGTCACACTTCACATGGACCTGTCCAT  ATGAATATCCTCCTTAGT |
| Del179_UP | CAGGGAGGGAACAGCAAAAC |
| Del180_P1 | CGTGAGCTCAGGAAAAAGCTACAACGTATTGAAGGTAATGACTCCAACGTG  TAGGCTGGAGCTGCTTC |
| Del180_P2 | CCCGGTACGCACCAGAAAATCATTGATATGGCCATGAATGGCGTTGGCATA  TGAATATCCTCCTTAGT |
| Del180_UP | AATTACGTGATGAGCGGCAG |
| Del181_P1 | CCATTTCCGCGCAGACGATGACGTCACTGCCCGGCTGTATGCGCGAGGGT GTAGGCTGGAGCTGCTTC |
| Del181_P2 | GGTGATGCTGCCAACTTACTGATTTAGTGTATGATGGTGTTTTTGAGCATAT  GAATATCCTCCTTAGT |
| Del181_UP | CGTCCTCCGTATCCTGTCAT |
| Del182_P1 | CTCCTTTCCTGAAAAGTCGGCGATAAATTGGTTACCAATATCTTCATAGTG  TAGGCTGGAGCTGCTTC |
| Del182_P2 | GGAAATAAATTAAGGTTGACCATGTCCGAAACTAAGCAGGCGTATTTACAT  ATGAATATCCTCCTTAGT |
| Del182_UP | CACCATTTCGTCCAAGCTCG |
| Del183_P1 | CAGTGCTAAAATAGCTGTAAATAAAAACAGTGTATTCAGGTGACCACC GT GTAGGCTGGAGCTGCTTC |
| Del183_P2 | CTTTAATCCATTTCGAAAGAAGTAATCCTCATTAGCGCCCGCAGGGGCGCA  TATGAATATCCTCCTTAGT |
| Del183_UP | CCGGATCGTTGTTGTAGTCG |
| Del184_P1 | CATGCGTGAGGCGCTGGATGAGTAGCGCCCCTGCGGGCGCTAATGAGGA GT GTAGGCTGGAGCTGCTTC |
| Del184_P2 | GGTGTGCATCGTGTTGTTACTCTCACTGGCAGCGGTGTGGATCAGTAA CAT  ATGAATATCCTCCTTAGT |
| Del184_UP | TTGACGCAGGTAAGAAAGCG |
| Del185_P1 | GCTCAGCGGCCGCTTAAAGTAATGGATTTCCAGCAATCCGACCGTCTTGGT  GTAGGCTGGAGCTGCTTC |
| Del185_P2 | GGTGCAGATCTTGGCATTTTCATTATGAGAAAGGTCTGAGGAGAAAAGCCA  TATGAATATCCTCCTTAGT |
| Del185_UP | ATAGCAAACCCGCACACATC |
| Del186_P1 | CTCATCAAGTTTCTGGCTGGTCGTGGAAATCCATTGCATGCTTTTCTCCGTG  TAGGCTGGAGCTGCTTC |
| Del186_P2 | 5GTTGAAGACGTAACGGTGTTGCGCTGCTTTATTGAAACCGGAGTGATGACA  TATGAATATCCTCCTTAGT |
| Del186_UP | GGTCCTCTATCGGGTCATGG |
| DelIS1b_P2 | GCGCCTTTCTTACATCGAGAAAAAGAAAGGCCGCCAGACGACGCATATCA  TATGAATATCCTCCTTAGT |
| Del Inc_P1 | CCTCAAAAACACCATCATACACTAAATCAGTAAGTTGGCACCATTACCGTGT  AGGCTGGAGCTGCTTC |
| Del Inc_P2  Del Inc_Down | CATATCGTATTTTCCTTCCGTGATATAACCTCCATGACGATTGAATAGCATA  TGAATATCCTCCTTAGT  GACACCAATGCGCCTTTCTT |
| RepECtermFor | AGAAGGAGATATAACTATGGCGGAAATAGCGGTTATAAACCATAAAAAAC |
| RepECtermRev | GTGGTGGTGATGGTGATGGCCTTCAATCGTCATGGAGGTTATATCACGG |
| incCFor | CAGGGAGGGAACAGCAAAAC |
| incCRev | CCGTGATATAACCTCCATGACG |
