# Supplementary material for: The incC Sequence Is Required for R27 Plasmid Stability
Source: Front Microbiol. 2016 May 3;7:629. doi: 10.3389/fmicb.2016.00629 (PMC4853401; doi:10.3389/fmicb.2016.00629)
Supplement: TABLE S2 — R27 sequences included in the different deletions. [file Table_2.DOCX]

| **Deletion** | | | **Start** | | | **End** | | **Lenght (bp)** | **Genes** |
| --- | --- | --- | --- | --- | --- | --- | --- | --- | --- |
| **Del1** | | | 40.355 | | | 46.955 | | 6.600 | **R0037**: 28.03 pct identical (3 gaps) to 132 residues of an approx. 240 aa protein - P120 [Mycoplasma hominis]  **R0038**: 39.29 pct identical (0 gaps) to 28 residues of an approx. 1976 aa protein - non-muscle myosin heavy chain [Drosophila melanogaster]  **R0039**: 39.47 pct identical (0 gaps) to 38 residues of an approx. 272 aa protein - tRNA delta(2)-isopentenylpyrophosphate transferase (miaA) [Helicobacter pylori]  **R0040**: 23.61 pct identical (3 gaps) to 72 residues of an approx. 520 aa protein - ATP synthase F1 alpha subunit [Escherichia coli]  **R0041**: 37.41 pct identical (10 gaps) to 270 residues of an approx. 288 aa protein - DNA adenine methylase (dam). [Haemophilus influenzae]  **R0042**: 29.17 pct identical (0 gaps) to 72 residues of an approx. 288 aa protein - Chromosome Partitioning Protein [Chlamydia trachomatis]  **R0043**: 70.92 pct identical (3 gaps) to 557 residues of an approx. 568 aa protein - restriction methylase [Serratia marcescens] |
| **Del2** | | | 46.955 | | | 53.655 | | 6.700 | **R0043**  **R0044**: 34.09 pct identical (0 gaps) to 44 residues of an approx. 1632 aa protein - exabrachion [Homo sapiens]  **R0045**: 26.23 pct identical (1 gap) to 61 residues of an approx. 1968 aa protein - Fat protein [Synechocystis sp.]  **R0046**: IS30 transposase; 100.00 pct identical amino acid sequence and equal length to IS30 transposase [Escherichia coli]  **R0047**: 56.00 pct identical (0 gaps) to 25 residues of an approx. 520 aa protein - aldehyde dehydrogenase [Pichia angusta]  **R0048**: 31.58 pct identical (0 gaps) to 38 residues of an approx. 288 aa protein - hypothetical protein [Aquifex aeolicus]  **R0049**: 27.78 pct identical (1 gap) to 72 residues of an approx. 280 aa protein - inner membrane protein MalG [Thermococcus litoralis] |
|  | |  | |  |  | |  |  |  |
|  |  |  |  |  |  |  |  |  |  |
| **Del3** | | | 53.655 | | | 60.355 | | 6.700 | **R0049**  **R0050**: repHIB  **R0051**: 29.73 pct identical (3 gaps) to 74 residues of an approx. 904 aa protein - RNA polymerase II largest subunit {C-terminal} [Dictyostelium discoideum]  **R0052**: 31.91 pct identical (0 gaps) to 47 residues of an approx. 712 aa protein - acid-rich protein [Caenorhabditis elegans]  **R0053**: 2.43 pct identical (0 gaps) to 37 residues of an approx. 216 aa protein - ras related protein Rab5b [Homo sapiens]  **R0054**: 36.07 pct identical (2 gaps) to 61 residues of an approx. 1056 aa protein - PPE [Mycobacterium tuberculosis]  **R0055**: 20.71 pct identical (3 gaps) to 140 residues of an approx. 1744 aa protein - hypothetical protein [Synechocystis sp.]  **R0056**: 44.83 pct identical (0 gaps) to 29 residues of an approx. 464 aa protein - surfactin synthetase subunit [Bacillus subtilis] |
| **Del4** | | | 60.355 | | | 67.055 | | 6.700 | **R0057**: 29.73 pct identical (3 gaps) to 111 residues of an approx. 2120 aa protein - myosin heavy chain [Dictyostelium discoideum]  **R0058**: 48.28 pct identical (1 gap) to 29 residues of an approx. 608 aa protein - PbTRAP [Plasmodium berghei]  **R0059**: 33.33 pct identical (1 gap) to 39 residues of an approx. 232 aa protein - S-crystallin [Loligo opalescens]  **R0060**: 0.69 pct identical (5 gaps) to 101 residues of an approx. 640 aa protein - K5 lyase [coliphage K5]  **R0061**: 23.26 pct identical (0 gaps) to 86 residues of an approx. 488 aa protein - hypothetical protein Rv2434c [Mycobacterium tuberculosis]  **R0062**: 38.71 pct identical (0 gaps) to 31 residues of an approx. 424 aa protein predicted using Genefinder; Similarity to Yeast mitochondrial [Caenorhabditis elegans]  **R0063**: 2.61 pct identical (0 gaps) to 46 residues of an approx. 536 aa protein - hypothetical protein [Schizosaccharomyces pombe]  **R0064**: 23.64 pct identical (0 gaps) to 55 residues of an approx. 344 aa protein - probable glycosyl transferase [Vibrio cholerae]  **R0065**: 30.10 pct identical (4 gaps) to 103 residues of an approx. 312 aa protein - hypothetical protein [Synechocystis sp.] |
| **Del5** | | | 67.055 | | | 74.086 | | 7.031 | **R0066**: 37.50 pct identical (1 gap) to 48 residues of an approx. 1320 aa protein - similar in the amino-half to peroxidases and in the carboxy-half to [Caenorhabditis elegans]  R0067: 38.71 pct identical (0 gaps) to 31 residues of an approx. 584 aa protein - Fln29 [Homo sapiens]  R0068: 25.35 pct identical (0 gaps) to 71 residues of an approx. 1352 aa protein - vascular endothelial growth factor receptor [Coturnix coturnix]  **R0069**: 31.43 pct identical (0 gaps) to 35 residues of an approx. 520 aa protein - endoglucanase [Clostridium cellulovorans]  **R0070**: 38.18 pct identical (3 gaps) to 55 residues of an approx. 440 aa protein - unknown [Homo sapiens]  R0071: 36.59 pct identical (0 gaps) to 41 residues of an approx. 712 aa protein - UNC-93 protein [Caenorhabditis elegans]  **R0072**: 39.47 pct identical (0 gaps) to 38 residues of an approx. 344 aa protein - NS1-OspA [Borrelia burgdorferi]  **R0073**: 60.71 pct identical (0 gaps) to 28 residues of an approx. 224 aa protein - unknown [Bacteriophage Mx8]  **R0074**: 27.42 pct identical (1 gap) to 62 residues of an approx. 464 aa protein - unknown [Bacteriophage Mx8]  **R0075**: 31.11 pct identical (0 gaps) to 45 residues of an approx. 160 aa protein - olfactory receptor protein [Necturus maculosus] |
| **Del6** | | | 83.018 | | | 90.065 | | 7.047 | **R0086**: 28.89 pct identical (9 gaps) to 90 residues of an approx. 376 aa protein - mannosyltransferase B [Aquifex aeolicus]  **R0087**: 28.57 pct identical (1 gap) to 77 residues of an approx. 440 aa protein - amino acid transporter [Methanococcus jannaschii]  **R0088**: 46.94 pct identical (0 gaps) to 49 residues of an approx. 72 aa protein - L0009 (LEE element) [Escherichia coli]  **R0089**: 40.00 pct identical (0 gaps) to 40 residues of an approx. 1136 aa protein - receptor-associated tyrosine kinase [Homo sapiens]  R0090: 44.83 pct identical (0 gaps) to 29 residues of an approx. 520 aa protein - RNA polymerase II associated protein RAP74 [Homo sapiens]  **R0091**: 42.42 pct identical (0 gaps) to 33 residues of an approx. 456 aa protein - ATP Binding Protein [Bacillus subtilis]  **R0092**: 26.83 pct identical (0 gaps) to 41 residues of an approx. 760 aa protein - hypothetical protein [Schizosaccharomyces pombe]  **R0093**: 40.54 pct identical (0 gaps) to 37 residues of an approx. 1832 aa protein - contains multiple regions of similarity to EGF-type repeats [Caenorhabditis elegans]  **R0094**: insB; 98.97 pct identical (0 gaps) to 97 residues of an approx. 152 aa protein - transposase [Escherichia coli]  **R0095**: insA; 100.00 pct identical amino acid sequence and equal length to insA peptide [Escherichia coli]  **R0096**: 99.05 pct identical to 316 amino acids of corA gene product (magnesium and cobalt transport protein)[Escherichia coli]  **R0097**: 100.00 pct identical (0 gaps) to 93 residues of an approx. 168 aa protein - f161 [Escherichia coli]  **R0098**: 29.85 pct identical (2 gaps) to 67 residues of an approx. 792 aa protein - similar to ribonucleoside-diphosphate reductase (alpha subunit)[Bacillus subtilis]  **R0099**: 32.20 pct identical (1 gap) to 59 residues of an approx. 312 aa protein - mitochondrial carrier protein-like; similar to Q09461 [Homo sapiens] |
| **Del7** | | | 90.001 | | | 96.150 | | 6.149 | **R0099**  **R0100**: 22.64 pct identical (0 gaps) to 106 residues of an approx. 240 aa protein orf - hypothetical protein [Escherichia coli]  **R0101**: 73.08 pct identical to 52 amino acids of an approx. 56 aa protein - flmA gene product [Plasmid F] (function= putative plasmid functions; plasmid maintenance, copy number)  **R0102**: 31.37 pct identical (1 gap) to 51 residues of an approx. 488 aa protein -atrolysin e [Trimeresurus mucrosquamatus]  **R0103**: 30.88 pct identical (0 gaps) to 68 residues of an approx. 960 aa protein - kinesin-like protein [Leishmania donovani]  **R0104**: 38.33 pct identical (2 gaps) to 60 residues of an approx. 296 aa protein - ParB [Caulobacter crescentus]  **R0105**: 32.56 pct identical (1 gap) to 43 residues of an approx. 312 aa protein - ipothetical protein Rv1695 [Mycobacterium tuberculosis]  **R0106**: 37.93 pct identical (0 gaps) to 29 residues of an approx. 792 aa protein - muscarinic acetylcholine receptor [Drosophila melanogaster]  **R0107**: 43.33 pct identical (0 gaps) to 30 residues of an approx. 240 aa protein - o238 [Escherichia coli]  **R0108**: 34.69 pct identical (0 gaps) to 49 residues of an approx. 328 aa protein - G-protein coupled receptor protein [Saimiriine herpesvirus]  **R0109**: 36.00 pct identical (3 gaps) to 75 residues of an approx. 376 aa protein - lignin peroxidase [Phanerochaete chrysosporium]  **R0110**: 26.98 pct identical (1 gap) to 63 residues of an approx. 184 aa protein - unknown protein [Synechocystis sp.] |
| **Del8** | | | 96.151 | | | 101.814 | | 5.663 | **R0111**: 7.31 pct identical (4 gaps) to 67 residues of an approx. 80 aa protein orf - hypothetical protein [Escherichia coli]  **R0112**: 45.83 pct identical (0 gaps) to 48 residues of an approx. 576 aa protein - epidermal keratin subunit I [Mus musculus]  **R0113**: 20.00 pct identical (0 gaps) to 45 residues of an approx. 496 aa protein - pantothenate permease (panF-1) [Archaeoglobus fulgidus]  **R0114**: 23.19 pct identical (2 gaps) to 69 residues of an approx. 568 aa protein homology with proteasome component C5; coded for by C. Elegans [Caenorhabditis elegans]  **R0115**: 57.14 pct identical (0 gaps) to 21 residues of an approx. 296 aa protein - unknown [Methanobacterium thermoautotrophicum]  **R0116**: 30.07 pct identical (4 gaps) to 143 residues of an approx. 288 aa protein - proteinase IV [Aquifex aeolicus]  **R0117**: 27.54 pct identical (0 gaps) to 69 residues of an approx. 568 aa protein - NADH dehydrogenase subunit 2 [Mitochondrion Pichia]  **R0118**: 35.29 pct identical (0 gaps) to 51 residues of an approx. 1928 aa protein similar to phosphoinositide-specific phospholipase C; cDNA EST [Caenorhabditis elegans] |
| **Del9** | | | 113.290 | | | 120.030 | | 6.740 | **R0128**: 22.50 pct identical (11 gaps) to 280 residues of an approx. 2384 aa -protein partial CDS [Caenorhabditis elegans]  **R0129**: 25.00 pct identical (0 gaps) to 60 residues of an approx. 528 aa protein - inosine 5 [Streptomyces coelicolor]  **R0130**: bfpH; 31.01 pct identical (2 gaps) to 129 residues of an approx. 216 aa protein - TrbN [Enterobacter aerogenes] (function = Cell envelope: putative outer membrane constituents)  R0131: dsbC; 36.41 pct identical (10 gaps) to 217 residues of an approx. 240 aa protein - dsbC [Erwinia chrysanthemi] (function = putative enzyme; central intermediary metabolism; disulphide)  **R0132**: 22.77 pct identical (2 gaps) to 101 residues of an approx. 712 aa protein - DNA43 [Saccharomyces cerevisiae] |
| **Del10** | | | 120.031 | | | 125.077 | | 5.046 | **R0133**: 8.57 pct identical (0 gaps) to 56 residues of an approx. 328 aa protein - protein-export membrane protein (secF)[Helicobacter pylori]  **R0134**: 30.99 pct identical (2 gaps) to 71 residues of an approx. 1512 aa protein - C26D10.4 [Caenorhabditis elegans]  **R0135**: 28.44 pct identical (20 gaps) to 225 residues of an approx. 256 aa protein - outer membrane protein [Coxiella burnetii]  **R0136**: mucB; 47.10 pct identical (1 gap) to 414 residues of an approx. 424 aa protein - mucB protein (AA 1-421) [Enterobacteriaceae Bacteria] (function = Macromolecule synthesis: DNA - replication,repair, restriction/modification)  **R0137**: mucA; 58.20 pct identical (0 gaps) to 122 residues of an approx. 152 aa protein – MucA [Morganella morganii] (function = Macromolecule synthesis: DNA - replication, repair, restriction/modification)  **R0138**: 23.75 pct identical (0 gaps) to 80 residues of an approx. 584 aa protein - leucine-rich repeat protein SHOC-2 [Homo sapiens]  **R0139**: 26.32 pct identical (1 gap) to 76 residues of an approx. 416 aa protein - antigen NY-CO-8 [Homo sapiens] |
| **Del11** | | | 125.078 | | | 131.855 | | 6.777 | **R0140:**22.98 pct identical (0 gaps) to 161 residues of an approx. 4688 aa protein - plectin [Rattus norvegicus]  **R0141**: 35.56 pct identical (0 gaps) to 45 residues of an approx. 344 aa protein - N-acetylmuramoyl-L-alanine amidase [Bacteriophage A511]  **R0142**: 34.69 pct identical (2 gaps) to 49 residues of an approx. 792 aa protein - no definition line [Drosophila melanogaster]  **R0143**: 30.88 pct identical (2 gaps) to 68 residues of an approx. 760 aa protein – gelsolin [Homarus americanus]  **R0144**: citA; 99.30 pct identical amino acid sequence and equal length to citrate utilization protein A [Transposon Tn3411] (function = Transport of small molecules: Anions) |
| **Del12** | | | 131.856 | | | 138.431 | | 6.575 | **R0145**: citB; 99.21 pct identical to 379 amino acids of citrate utilization protein B [Transposon Tn3411]  **R0146**: 37.04 pct identical (38 gaps) to 459 residues of an approx. 832 aa protein - hypothetical protein [Rhodobacter capsulatus]  **R0147**: nac; 36.03 pct identical (7 gaps) to 247 residues of an approx. 312 aa protein - Nitrogen assimilation regulatory protein NAC [Escherichia coli] (function = Central intermediary metabolism: putative regulator)  **R0148**: 47.74 pct identical (2 gaps) to 421 residues of an approx. 464 aa protein - Y4bF [Rhizobium sp.] (function = External elements : IS and transposon functions; DNA excision and insertion)  **R0149**: 27.40 pct identical (4 gaps) to 73 residues of an approx. 616 aa protein - HSP70 [Staphylococcus aureus] |
| **Del13** | | | 138.432 | | | 144.479 | | 6.047 | **R0150**:  34.15 pct identical (0 gaps) to 41 residues of an approx. 840 aa protein - Unknown protein [Arabidopsis thaliana]  **R0151**: 24.77 pct identical (5 gaps) to 109 residues of an approx. 440 aa protein - A. fulgidus predicted coding region AF1180 [Archaeoglobus fulgidus]  **R0152**: 34.04 pct identical (0 gaps) to 47 residues of an approx. 4296 aa protein - polycystic kidney disease 1 protein [Mus musculus]  **R0153**: parB; 39.87 pct identical (2 gaps) to 158 residues of an approx. 184 aa protein - nuclease, putative [Haemophilus influenzae]  **R0154**: 66.74 pct identical (0 gaps) to 442 residues of an approx. 480 aa protein - EcoRII cytosine methylase (AA 1-477) [Escherichia coli]  **R0155**: 36.11 pct identical (0 gaps) to 36 residues of an approx. 576 aa protein - Strong similarity to Arabidopsis oligopeptide transporter [Arabidopsis thaliana]  **R0156**: 24.00 pct identical (10 gaps) to 325 residues of an approx. 456 aa protein - unknown [Sphingomonas aromaticivorans]  **R0157**: 34.48 pct identical (0 gaps) to 58 residues of an approx. 296 aa protein - hypothetical protein [Borrelia burgdorferi] |
| **Del14** | | | 144.480 | | | 150.565 | | 6.085 | **R0158**: tlpA; 23.56 pct identical (3 gaps) to 208 residues of an approx. 1096 aa protein - ORF 73, contains large complex repeat CR 73. [Kaposi's sarcoma-associated]  **R0159**:  37.21 pct identical (0 gaps) to 43 residues of an approx. 512 aa protein - B. burgdorferi predicted coding region BB0038. [Borrelia burgdorferi]  **R0160**: 33.33 pct identical (0 gaps) to 57 residues of an approx. 608 aa protein - lti65 [Arabidopsis thaliana]  **R0161**: 32.20 pct identical (0 gaps) to 59 residues of an approx. 752 aa protein - DNA inversion product [Escherichia coli]  **R0162**: 31.15 pct identical (0 gaps) to 61 residues of an approx. 1152 aa protein - BRCA1 [Cynocephalus variegatus]  **R0163**: 50.00 pct identical (0 gaps) to 20 residues of an approx. 120 aa protein - hypothetical protein [Synechocystis sp.]  **R0164**: H-NS; 61.94 pct identical amino acid sequence and equal length to - H-NSB [Escherichia coli]  **R0165**: 31.48 pct identical (0 gaps) to 54 residues of an approx. 376 aa protein - sac operon related regulation protein [Bacillus sp.]  **R0166**: 44.44 pct identical (1 gap) to 36 residues of an approx. 448 aa protein - iron-molybdenum cofactor biosynthesis subunit [Herbaspirillum seropedicae]  **R0167**: 36.17 pct identical (1 gap) to 47 residues of an approx. 328 aa protein - orf1 [Rhodococcus erythropolis] |
| **Del15** | | | 150.566 | | | 156.499 | | 5.933 | **R0168**:  34.62 pct identical (2 gaps) to 78 residues of an approx. 600 aa protein - neoxanthin cleavage enzyme-like protein [Arabidopsis thaliana]  **R0169**: 45.71 pct identical (0 gaps) to 35 residues of an approx. 584 aa protein - similar to malate dehydrogenase [Bacillus subtilis]  **R0171**: 55.56 pct identical (0 gaps) to 18 residues of an approx. 304 aa protein - hypothetical protein Rv1515c [Mycobacterium tuberculosis]  **R0172**: 36.67 pct identical (0 gaps) to 30 residues of an approx. 488 aa protein - NAD-linked malic enzyme; malate oxidoreductase [Escherichia coli]  **R0173**: 32.26 pct identical (2 gaps) to 62 residues of an approx. 984 aa protein - 976aa long hypothetical protein [Pyrococcus horikoshii]  **R0174**: 35.56 pct identical (1 gap) to 45 residues of an approx. 280 aa protein - diaminopimelate epimerase [Synechocystis sp.]  **R0175**: 24.44 pct identical (1 gap) to 45 residues of an approx. 328 aa protein - transcription factor [Lactococcus lactis]  **R0176**: 34.88 pct identical (0 gaps) to 43 residues of an approx. 480 aa protein - astacus egg astacin [Astacus astacus]  **R0177**: insB; 93.41 pct identical amino acid sequence and equal length to insB [Escherichia coli]  **R0178**: insA; 00.00 pct identical amino acid sequence and equal length to insA gene product [Escherichia coli] |
| **Del16** | | | 156.500 | | | 163.000 | | 6.500 | **R0179**: RepF1B;  92.15 pct identical to 256 amino acids of Protein E [Plasmid F]  **R0180**: insB; 94.01 pct identical amino acid sequence and equal length to insB [Escherichia coli]  **R0181**: insA; 96.39 pct identical to 83 residues of a 91 aa protein - insA gene product [Escherichia coli]  **R0182**: Hha; 48.48 pct identical (0 gaps) to 66 residues of an approx. 80 aa protein - Hha protein [Escherichia coli]  **R0183**: 42.86 pct identical (0 gaps) to 56 residues of an approx. 344 aa protein - ORF2, put. cre protein (aa 1-343) [Bacteriophage P1]  **R0184**: 28.95 pct identical (0 gaps) to 38 residues of an approx. 856 aa protein - hypothetical protein [Arabidopsis thaliana]  **R0185**: 32.81 pct identical (0 gaps) to 64 residues of an approx. 360 aa protein - CheB [Bacillus subtilis]  **R0186**: 26.13 pct identical (6 gaps) to 111 residues of an approx. 312 aa protein - DNA replication terminus site-binding protein [Klebsiella pneumoniae] |
| **Del17** | | | 163.001 | | | 168.600 | | 5.599 | **R0187**: 52.17 pct identical (0 gaps) to 23 residues of an approx. 1552 aa protein - unnamed protein product [Homo sapiens]  **R0188**: 29.73 pct identical (0 gaps) to 37 residues of an approx. 432 aa protein – allergen [Aspergillus fumigatus]  **R0189**: 35.29 pct identical (1 gap) to 51 residues of an approx. 344 aa protein - C38C6.4 [Caenorhabditis elegans]  **R0190**: 35.09 pct identical (1 gap) to 57 residues of an approx. 440 aa protein - COT1 protein [Saccharomyces cerevisiae]  **R0191**: 29.03 pct identical (1 gap) to 62 residues of an approx. 2152 aa protein - protein phosphatase 1 catalytic subunit [Trypansoma brucei]  **R0192**: 44.12 pct identical (0 gaps) to 34 residues of an approx. 328 aa protein - branched-chain amino acid abc transporter, permease protein (brae-2) [Archaeoglobus fulgidus]  **R0193**: 35.90 pct identical (0 gaps) to 39 residues of an approx. 768 aa protein - unnamed protein product [Arabidopsis thaliana]  **R0194**: 41.67 pct identical (2 gaps) to 36 residues of an approx. 1488 aa protein – cystic fibrosis transmembrane conductance regulator [Xenopus laevis]  **R0195**: 100.00 pct identical amino acid sequence and equal length to IS2 ORF; alternate gene name yi22 [Escherichia coli]  **R0197**: 100.00 pct identical amino acid sequence and equal length to IS2 ORF; alternate gene name yi21 [Escherichia coli]  **R0198**: 50.00 pct identical (0 gaps) to 24 residues of an approx. 376 aa protein - putative protein. [Arabidopsis thaliana] |
| **Del18** | | | 168.601 | | | 175.050 | | 6.449 | **R0199**: 38.10 pct identical (1 gap) to 42 residues of an approx. 1248 aa protein - YOR50-17 [Saccharomyces cerevisiae]  **R0200**:  38.46 pct identical (2 gaps) to 52 residues of an approx. 320 aa protein - Nmi [Mus musculus]  **R0201**: 4.51 pct identical (1 gap) to 102 residues of an approx. 432 aa protein - Beta-Ketoacyl synthase/Acyl transferase [Streptomyces coelicolor]  **R0202**: 23.08 pct identical (1 gap) to 78 residues of an approx. 2872 aa protein - reticulocyte binding protein 1 [Plasmodium vivax]  **R0203**: 27.78 pct identical (3 gaps) to 90 residues of an approx. 520 aa protein - RNA synthesis factor (drpA) [Escherichia coli]  **R0204**: 31.08 pct identical (2 gaps) to 74 residues of an approx. 184 aa protein - UmoA [Proteus mirabilis]  **R0205**: 34.15 pct identical (0 gaps) to 41 residues of an approx. 1336 aa protein agrin [Discopyge ommata] |
| **Del19** | | | 175.051 | | | 180.461 | | 5.410 | **R0206**: 1.78 pct identical (6 gaps) to 129 residues of an approx. 800 aa protein - ORF 48; EDLF5; sim. to EBV BRRF2 [Saimiriine herpesvirus]  **R0207**: 25.00 pct identical (0 gaps) to 64 residues of an approx. 424 aa protein - DNA gyrase beta-subunit [Shewanella putrefaciens]  **R0208**: 30.51 pct identical (2 gaps) to 59 residues of an approx. 5160 aa protein - peptide synthetase [Metarhizium anisopliae]  **R0209**: 30.84 pct identical (0 gaps) to 107 residues of an approx. 280 aa protein - NirQ protein [Pseudomonas stutzeri]  **R0210**: 21.92 pct identical (1 gap) to 73 residues of an approx. 1664 aa protein – vitellogenin [Oncorhynchus mykiss] |
| **Del 179** | | | 156.848 | | | 157.603 | | 755 | **R0179**: RepF1B;  92.15 pct identical to 256 amino acids of Protein E [Plasmid F] |
| **Del180** | | | 158.185 | | | 158.688 | | 503 | **R0180**: insB; 94.01 pct identical amino acid sequence and equal length to insB [Escherichia coli] |
| **Del 181** | | | 158.607 | | | 158.891 | | 284 | **R0181**: insA; 96.39 pct identical to 83 residues of a 91 aa protein - insA gene product [Escherichia coli] |
| **Del 182** | | | 159.190 | | | 159.375 | | 185 | **R0182**: Hha; 48.48 pct identical (0 gaps) to 66 residues of an approx. 80 aa protein - Hha protein [Escherichia coli] |
| **Del 183** | | | 159.617 | | | 160.798 | | 1181 | **R0183**: 42.86 pct identical (0 gaps) to 56 residues of an approx. 344 aa protein - ORF2, put. cre protein (aa 1-343) [Bacteriophage P1] |
| **Del 184** | | | 160.823 | | | 161.167 | | 344 | **R0184**: 28.95 pct identical (0 gaps) to 38 residues of an approx. 856 aa protein - hypothetical protein [Arabidopsis thaliana] |
| **Del 185** | | | 161.103 | | | 162.011 | | 908 | **R0185**: 32.81 pct identical (0 gaps) to 64 residues of an approx. 360 aa protein - CheB [Bacillus subtilis] |
| **Del 186** | | | 162.022 | | | 162.981 | | 959 | **R0186**: 26.13 pct identical (6 gaps) to 111 residues of an approx. 312 aa protein - DNA replication terminus site-binding protein [Klebsiella pneumoniae] |
| **Del 179-181 (A)** | | | 156.848 | | | 158.891 | | 2043 | **R0179**: RepF1B;  92.15 pct identical to 256 amino acids of Protein E [Plasmid F]  **R0180**: insB; 94.01 pct identical amino acid sequence and equal length to insB [Escherichia coli]  **R0181**: insA; 96.39 pct identical to 83 residues of a 91 aa protein - insA gene product [Escherichia coli] |
| **Del 182-183 (C)** | | | 159.190 | | | 160.798 | | 1608 | **R0182**: Hha; 48.48 pct identical (0 gaps) to 66 residues of an approx. 80 aa protein - Hha protein [Escherichia coli]  **R0183**: 42.86 pct identical (0 gaps) to 56 residues of an approx. 344 aa protein - ORF2, put. cre protein (aa 1-343) [Bacteriophage P1] |
| **Del 183-184 (E)** | | | 159.617 | | | 161.167 | | 1550 | **R0183**: 42.86 pct identical (0 gaps) to 56 residues of an approx. 344 aa protein - ORF2, put. cre protein (aa 1-343) [Bacteriophage P1]  **R0184**: 28.95 pct identical (0 gaps) to 38 residues of an approx. 856 aa protein - hypothetical protein [Arabidopsis thaliana] |
| **Del182-184 (D)** | | | 159.190 | | | 161.167 | | 1977 | **R0182**: Hha; 48.48 pct identical (0 gaps) to 66 residues of an approx. 80 aa protein - Hha protein [Escherichia coli]  **R0183**: 42.86 pct identical (0 gaps) to 56 residues of an approx. 344 aa protein - ORF2, put. cre protein (aa 1-343) [Bacteriophage P1]  **R0184**: 28.95 pct identical (0 gaps) to 38 residues of an approx. 856 aa protein - hypothetical protein [Arabidopsis thaliana] |
| **Del185-186 (G)** | | | 161.103 | | | 162.981 | | 1878 | **R0185**: 32.81 pct identical (0 gaps) to 64 residues of an approx. 360 aa protein - CheB [Bacillus subtilis  **R0186**: 26.13 pct identical (6 gaps) to 111 residues of an approx. 312 aa protein - DNA replication terminus site-binding protein [Klebsiella pneumoniae] |
| **Del179-182 (B)** | | | 156.848 | | | 159.375 | | 2527 | **R0179**: RepF1B;  92.15 pct identical to 256 amino acids of Protein E [Plasmid F]  **R0180**: insB; 94.01 pct identical amino acid sequence and equal length to insB [Escherichia coli]  **R0181**: insA; 96.39 pct identical to 83 residues of a 91 aa protein - insA gene product [Escherichia coli]  **R0182**: Hha; 48.48 pct identical (0 gaps) to 66 residues of an approx. 80 aa protein - Hha protein [Escherichia coli] |
| **Del183-186 (F)** | | | 159.617 | | | 162.981 | | 3364 | **R0183**: 42.86 pct identical (0 gaps) to 56 residues of an approx. 344 aa protein - ORF2, put. cre protein (aa 1-343) [Bacteriophage P1]  **R0184**: 28.95 pct identical (0 gaps) to 38 residues of an approx. 856 aa protein - hypothetical protein [Arabidopsis thaliana]  **R0185**: 32.81 pct identical (0 gaps) to 64 residues of an approx. 360 aa protein - CheB [Bacillus subtilis  **R0186**: 26.13 pct identical (6 gaps) to 111 residues of an approx. 312 aa protein - DNA replication terminus site-binding protein [Klebsiella pneumoniae] |
