# Supplementary material for: The incC Sequence Is Required for R27 Plasmid Stability
Source: Front Microbiol. 2016 May 3;7:629. doi: 10.3389/fmicb.2016.00629 (PMC4853401; doi:10.3389/fmicb.2016.00629)
Supplement: TABLE S3 — Results of the BlastN analysis. [file Table_3.DOCX]

BLAST: AF250878 (seq. 156.526-156.847)

Sequences producing significant alignments

Selected:31

| **Description** | **Max score** | **Total score** | **Query cover** | **E value** | **Ident** | **Accession** |
| --- | --- | --- | --- | --- | --- | --- |
| Salmonella enterica strain B71 plasmid pB71, complete sequence | 595 | 1301 | 100% | 1e-166 | 100% | KP899806.1 |
| Salmonella enterica strain 109/9 plasmid p109/9, complete sequence | 595 | 1301 | 100% | 1e-166 | 100% | KP899805.1 |
| Salmonella enterica strain F8475 plasmid pF8475, complete sequence | 595 | 1301 | 100% | 1e-166 | 100% | KP899804.1 |
| Escherichia coli strain 63743 plasmid pEQ2, complete sequence | 595 | 1301 | 100% | 1e-166 | 100% | NC_023277.2 |
| Escherichia coli strain T23 plasmid pEQ1, complete sequence | 595 | 1301 | 100% | 1e-166 | 100% | NC_023289.2 |
| Salmonella enterica subsp. enterica serovar Typhi str. P-stx-12 plasmid, complete sequence | 595 | 1301 | 100% | 1e-166 | 100% | CP003279.1 |
| Escherichia coli O111:H- str. 11128 plasmid pO111_1 DNA, complete sequence | 595 | 1301 | 100% | 1e-166 | 100% | AP010961.1 |
| Salmonella typhi R27 plasmid complete sequence | 595 | 1301 | 100% | 1e-166 | 100% | AF250878.1 |
| Salmonella enterica subsp. enterica serovar Typhi str. CT18 plasmid pHCM1 | 595 | 1301 | 100% | 1e-166 | 100% | AL513383.1 |
| Salmonella enterica Paratyphi A IncH1 multiple drug resistance, serovar Paratyphi A | 496 | 979 | 100% | 1e-136 | 100% | AM412236.1 |
| Plasmid R27 replication region | 379 | 1085 | 63% | 1e-101 | 100% | Y00547.1 |
| Klebsiella pneumoniae strain Kpn-3002cz plasmid pS-300cz, complete sequence | 350 | 601 | 100% | 1e-92 | 96% | KJ958927.1 |
| Klebsiella pneumoniae strain 7433 plasmid pTR2, complete sequence | 350 | 601 | 100% | 1e-92 | 96% | KJ187752.1 |
| Klebsiella pneumoniae strain blaNDM-1 plasmid plasmid1, complete sequence | 350 | 601 | 100% | 1e-92 | 96% | CP009116.1 |
| Klebsiella pneumoniae subsp. pneumoniae PittNDM01 plasmid3, complete sequence | 350 | 601 | 100% | 1e-92 | 96% | CP006801.1 |
| Klebsiella pneumoniae ATCC BAA-2146 plasmid pHg, complete sequence | 350 | 601 | 100% | 1e-92 | 96% | CP006662.1 |
| Klebsiella pneumoniae strain NK245 plasmid pK245, complete sequence | 350 | 601 | 100% | 1e-92 | 96% | DQ449578.1 |
| Klebsiella pneumoniae subsp. pneumoniae KPNIH27 plasmid pKPN-b0b, complete sequence | 322 | 579 | 92% | 3e-84 | 97% | CP007736.1 |
| Enterobacter asburiae strain CAV1043 plasmid pCAV1043-51, complete sequence | 311 | 632 | 99% | 5e-81 | 96% | CP011587.1 |
| Klebsiella pneumoniae str. Kp52.145, plasmid I, complete genome | 267 | 267 | 58% | 1e-67 | 92% | FO834904.1 |
| Klebsiella pneumoniae plasmid pKPoxa-48N2, complete sequence | 261 | 261 | 58% | 6e-66 | 92% | NC_021502.1 |
| Klebsiella oxytoca strain CAV1374 plasmid pCAV1374-228, complete sequence | 211 | 211 | 59% | 6e-51 | 87% | CP011634.1 |
| Klebsiella oxytoca KONIH1 plasmid pKOX-137, complete sequence | 211 | 211 | 59% | 6e-51 | 87% | CP008789.1 |
| Klebsiella pneumoniae strain NJ HT1872 plasmid pUSKPC3, complete sequence | 158 | 158 | 45% | 8e-35 | 86% | KJ721789.1 |
| Escherichia coli strain BK32533 plasmid pBK32533, complete sequence | 158 | 158 | 45% | 8e-35 | 86% | KP345882.1 |
| Escherichia coli strain MNCRE44 plasmid pMNCRE44_5, complete sequence | 158 | 158 | 45% | 8e-35 | 86% | CP010881.1 |
| Enterobacter cloacae strain 34978 plasmid p34978-139.941kb, complete sequence | 158 | 158 | 45% | 8e-35 | 86% | CP010363.1 |
| Klebsiella pneumoniae subsp. pneumoniae strain KPNIH32 plasmid pKPC-def, complete sequence | 158 | 158 | 45% | 8e-35 | 86% | CP009776.1 |
| Klebsiella pneumoniae subsp. pneumoniae strain KPNIH33 plasmid pKPC-63d, complete sequence | 158 | 158 | 45% | 8e-35 | 86% | CP009773.1 |
| Klebsiella pneumoniae strain BK30683 plasmid pBK30683, complete sequence | 158 | 158 | 45% | 8e-35 | 86% | KF954760.1 |
| Klebsiella pneumoniae strain BK30661 plasmid pBK30661, complete sequence | 158 | 158 | 45% | 8e-35 | 86% | KF954759.1 |
